# Supplementary material for: Green Contributions to the Chemistry of Perezone and Oxidation of the Double Bond of the Side Chain: A Theoretical Study and Cytotoxic Evaluation in MDA-MB231 Cells
Source: Molecules. 2025 Nov 30;30(23):4603. doi: 10.3390/molecules30234603 (PMC12693026; doi:10.3390/molecules30234603)
Supplement: Supplementary file 1 [file molecules-30-04603-s001.zip › molecules-3955571-supplementary.pdf]

Green Contributions to the Chemistry of Perezone and Oxidation of the Double Bond of the Side Chain: A Theoretical Study and Cytotoxic Evaluation in MDA-MB231 Cells

René Gerardo Escobedo-González [1,*], Joel Martínez [2], Adriana L. Rivera-Espejel [2], Claudia L. Vargas-Requena [3], María Inés Nicolás-Vázquez [2] and René Miranda Ruvalcaba [2,*]

* Correspondence: rene_escobedo@utcj.edu.mx (R.G.E.-G.); mirruv@comunidad.unam.mx (R.M.R.)

CONTENT

Table S1. Theoretical and experimental ^{1}H NMR chemical shifts of **3-10**.

Table S2. Theoretical and experimental ^{13}C NRM chemical shifts of **3-10**.

Table S3. Prediction of the absorption of isoperezone and its derivatives in different models.

Table S4. Results of the prediction of metabolism for isoperezone and its derivatives.

Structural characterization of compounds **3-10**.

Figure S1. Dose-response curves for cytotoxicity on breast cancer cells of derivatives **3-5**, **7**, **9-10**.

Figure S2. Optimized geometries for **3-6** with their respective stereocenter at C-12.

Figure S3. Optimized geometries for **7-10** with their respective stereocenter at C-12.

Figure S4. LUMO molecular orbital of the studied molecules.

Figure S5. Molecular electrostatic potential maps for stereoisomers with lower E_{LUMO} of compounds **3-10**.

Figure S6. RMSD for co-crystallized ligand Ac-DW3-KE (yellow) with respect to reference ligand at the crystal structures (gray) for illustrating good docking solution (RMSD ≤ 2.0 Å).

Table S1. Theoretical and experimental ^{1}H NMR chemical shift of **3-10**.

H	3			4			5			6			7			8			9			10		
	R	*S*	Exp	*R*	*S*	Exp	*R*	*S*	Exp	*R*	*S*	Exp	*R*	*S*	Exp	*R*	*S*	Exp	*R*	*S*	Exp	*R*	*S*	Exp
**H-3, OH-3**	6.90	6.83	7.25	6.82	6.36	6.47	6.89	7.00	3.17	6.29	6.26	6.46	6.89	7.00	7.20	6.74	6.26	6.43	7.08	6.91	6.75	6.59	6.28	6.48
**H-6, OH-6**	6.24	6.35	6.5	6.93	6.81	7.18	6.34	6.27	6.47	6.95	6.91	3.7	6.28	6.26	6.48	6.92	6.82	7.02	6.34	6.34	6.49	6.78	6.99	7.2
**H-7**	1.93	1.96	2.05	1.95	2.07	1.94	1.96	1.93	2.1	1.98	1.98	1.84	1.92	2.03	2.10	1.98	1.97	2.12	2.03	2.01	2.10	2.01	1.98	1.90
**H-8**	3.14	3.08	3.1	2.91	2.75	2.96	3.14	3.09	3.09	2.15	2.23	2.94	3.13	2.92	3.05	3.35	2.06	2.89	3.69	3.15	3.07	3.16	2.24	2.92
**H-9**	1.34	1.41	1.3	1.38	1.00	1.16	1.81	1.74	0.96	1.40	1.40	1.00	1.38	1.22	1.20	1.35	1.34	1.13	1.31	1.38	1.13	1.01	1.43	1.17
**H-10**	1.78	1.94	1.5	2.00	1.50	1.75	1.99	1.89	1.98	1.94	1.92	1.77	2.06	1.97	1.98	1.94	1.84	1.70	1.93	1.74	2.00	1.93	2.00	1.70
**H-11**	1.28	1.25	1.80	1.31	1.47	1.50	1.12	1.43	1.14	1.36	1.34	1.44	1.05	1.30	1.34	1.42	1.76	1.35	1.13	1.20	1.50	1.50	1.25	1.40
**H-12**	2.48	2.47	2.71	2.53	2.55	2.7	3.50	3.65	3.64	3.66	3.53	3.66	3.57	3.15	4.75	4.64	3.31	4.78	3.38	3.35	3.40	3.51	3.09	3.40
**OH-12**													5.04	3.69	4.83	1.16	1.70	4.81	1.91	1.11	7.25	4.05	1.18	7.50
**OH-13**																			2.87	2.63	7.25	1.47	1.20	7.50
**H-14**	1.21	1.14	1.24	1.20	1.26	1.24	1.21	1.19	1.21	0.90	1.04	1.16	1.00	1.28	1.14	1.50	1.50	1.17	1.02	0.96	1.16	1.33	1.03	1.21
**H-15**	0.97	1.05	1.21	1.08	1.20	1.21	0.77	1.05	1.21	1.14	0.94	1.17	1.33	1.50	2.06	1.11	1.60	1.17	0.78	1.04	1.22	1.38	1.28	1.23
**H-17**							1.28	1.22	1.27	1.26	1.34	1.37	1.984	2.018	2.18	1.93	1.77	1.19						
**H-18**							1.4303	1.3746	1.27	1.21	1.25	1.24												

Table S2. Theoretical and experimental ^{13}C NMR chemical shifts of **3-10**.

C	3			4			5			6			7			8			9			10		
	R	*S*	Exp	*R*	*S*	Exp	*R*	*S*	Exp	*R*	*S*	Exp	*R*	*S*	Exp	*R*	*S*	Exp	*R*	*S*	Exp	*R*	*S*	Exp
**C1**	196.4	195.6	187.3	192.6	195.9	188.4	196.4	196.4	188.4	191.7	192.0	188.4	195.6	195.0	187.3	192.5	192.5	188.0	195.7	196.3	184.2	191.8	191.9	188.3
**C2**	130.1	130.5	122.1	154.0	158.9	149.5	128.2	128.2	150.1	155.5	155.5	150.1	131.6	133.1	123.9	155.0	155.0	149.0	132.0	129.7	124.1	132.1	155.9	117.1
**C3**	160.0	159.2	151.2	146.8	156.4	133.1	162.0	162.0	133.3	143.1	143.0	133.3	159.2	157.1	151.2	146.2	146.2	133.2	157.2	159.4	151.1	137.2	142.8	133.1
**C4**	192.4	192.5	184.2	196.4	191.4	183.1	191.4	191.4	184.1	195.3	196.2	184.1	192.8	192.9	184.2	196.7	196.7	183.1	191.9	192.0	187.8	196.8	195.8	183.4
**C5**	148.3	147.5	140.8	122.3	123.1	116.9	149.6	149.6	116.8	122.6	123.0	116.8	147.6	147.7	140.7	122.2	122.2	116.9	148.4	148.2	140.9	137.3	122.2	149.4
**C6**	142.9	143.3	135.8	157.8	139.5	151.6	140.2	140.2	154.1	158.0	159.0	154.1	143.1	143.1	135.7	157.9	157.9	151.4	143.3	143.3	135.6	150.1	157.9	151.7
**C7**	17.7	17.8	14.7	10.5	10.7	7.8	19.0	19.0	7.8	10.3	10.6	7.8	17.7	18.2	14.7	10.9	10.9	7.8	17.9	18.2	14.0	12.1	10.6	7.9
**C8**	39.5	39.6	29.5	44.8	37.1	31.2	37.5	37.5	32.5	49.5	49.2	32.5	39.8	37.9	29.1	45.5	45.5	31.6	38.8	38.8	29.7	37.3	49.5	28.9
**C9**	22.6	22.8	18.0	23.0	22.5	18.0	23.3	23.3	23.2	23.7	23.5	23.2	22.6	18.4	18.2	22.9	22.9	18.7	20.9	22.7	23.0	26.4	23.6	19.6
**C10**	35.8	37.6	31.2	39.1	35.0	31.7	38.5	38.5	33.6	38.4	37.3	33.6	36.7	36.4	30.4	39.9	39.9	32.0	39.7	38.0	31.0	40.2	37.8	31.2
**C11**	33.6	34.4	26.4	30.4	33.0	23.1	30.9	30.9	27.4	33.6	34.5	27.4	36.7	36.1	27.9	31.5	31.5	27.1	32.5	36.3	28.7	32.5	34.1	26.5
**C12**	69.3	69.5	61.5	71.3	69.0	56.4	92.9	92.9	83.9	88.9	88.7	83.9	82.6	83.3	79.7	83.8	83.8	79.2	89.9	86.5	78.7	73.5	82.5	77.5
**C13**	62.8	62.3	49.0	62.1	63.4	49.0	88.5	88.5	80.5	87.6	86.9	80.5	100.3	96.5	72.3	95.7	95.7	72.3	77.3	78.0	77.3	73.9	79.5	72.5
**C14**	27.0	26.5	23.2	26.6	27.1	26.4	28.2	28.2	27.1	27.2	27.2	27.1	28.5	29.5	26.5	24.3	24.3	26.5	27.1	27.2	26.3	29.0	31.1	23.3
**C15**	19.6	20.8	18.4	20.6	20.7	19.6	23.6	23.6	19.7	24.4	24.3	19.7	19.7	25.0	24.9	22.6	22.5	25.2	22.8	22.9	18.2	19.3	22.8	18.8
**C16**							116.8	116.8	106.8	115.3	115.7	106.8	180.5	180.5	171.2	176.8	176.8	171.1						
**C17**							29.6	29.6	28.9	30.4	30.3	28.9	24.4	23.9	21.0	24.0	24.0	21.0						
**C18**							28.6	28.6	27.1	27.7	27.5	27.1												

Table S3. Prediction of the absorption of isoperezone and its derivatives in different models.

Absorption model	Molecules									
	**2**		**4**		**6**		**8**		**10**	
	Res [1]	P [2]	Res [1]	P [2]	Res [1]	P [2]	Res [1]	P [2]	Res [1]	P [2]
**Blood-brain barrier**	BBB+ [3]	0.798	BBB+ [3]	0.878	BBB+ [3]	0.903	BBB+ [3]	0.740	BBB+ [3]	0.718
**Human intestinal Absorption**	HIA+ [3]	0.995	HIA+ [3]	0.953	HIA+ [3]	0.927	HIA+ [3]	0.912	HIA+ [3]	0.913
**Caco-2 permeability**	Caco2+ [3]	0.720	Caco2+ [3]	0.604	Caco2- [4]	0.526	Caco2- [4]	0.561	Caco2+ [3]	0.500
**P-glycoprotein substrate**	NS [6]	0.522	S [5]	0.641	S [5]	0.538	S [5]	0.709	S [5]	0.767
**P-glycoprotein inhibitor**	I [7]	0.537	I [7]	0.580	I [7]	0.608	I [7]	0.514	NI [8]	0.787
	I [7]	0.644	I [7]	0.512	NI [8]	0.512	I [7]	0.579	NI [8]	0.750
**Renal Organic cation transporter**	NI [8]	0.850	NI [8]	0.878	NI [8]	0.861	NI [8]	0.905	NI [8]	0.909

[1] Results, [2] Probability, [3] Positive to absorption, [4] Negative to absorption, [5] Substrate, [6] No substrate, [7] Inhibitor, [8] No inhibitor.

Table S4. Results of the prediction of metabolism for isoperezone and its derivatives.

Metabolism model	Molecule									
	2		4		6		8		10	
	Res [1]	P [2]	Res [1]	P [2]	Res [1]	P [2]	Res [1]	P [2]	Res [1]	P [2]
**CYP450 2C9 Substrate**	NS [3]	0.822	NS [3]	0.801	NS [3]	0.8229	NS [3]	0.8720	NS [3]	0.8510
**CYP450 2D6 Substrate**	NS [3]	0.867	NS [3]	0.873	NS [3]	0.8880	NS [3]	0.9120	NS [3]	0.8892
**CYP450 3A4 Substrate**	S [4]	0.565	S [4]	0.648	S [4]	0.6304	S [4]	0.6520	S [4]	0.6797
**CYP450 1A2 Inhibitor**	NI [5]	0.686	NI [5]	0.784	NI [5]	0.7895	NI [5]	0.9288	NI [5]	0.8971
**CYP450 2C9 Inhibitor**	NI [5]	0.729	NI [5]	0.839	NI [5]	0.8618	NI [5]	0.8113	NI [5]	0.8284
**CYP450 2D6 Inhibitor**	NI [5]	0.802	NI [5]	0.907	NI [5]	0.9385	NI [5]	0.9295	NI [5]	0.9020
**CYP450 2C19 Inhibitor**	NI [5]	0.823	NI [5]	0.819	NI [5]	0.9075	NI [5]	0.8788	NI [5]	0.8067
**CYP450 3A4 Inhibitor**	NI [5]	0.883	NI [5]	0.759	NI [5]	0.7772	NI [5]	0.8516	NI [5]	0.7480

[1] Results, [2] Probability, [3] No Substrate, [4] Substrate, [5] No Inhibitor.

Structural characterization of compounds **3-10**

2-[4-(3,3-dimethyloxiran-2-yl)butan-2-yl]-3-hydroxy-5-methylcyclohexa-2,5-diene-1,4-dione (**3**): yellow solid was obtained with 85.5% isolated yield; mp 78-80 °C; ^{1}H NMR ($CDCl_3$/TMS) (δ/ppm): 7.25 (bs, 1H, OH), 6.50 (s, 1H, H-6), 3.10 (m, 1H, H-8), 2.71 (t, 1H, H-12), 2.05 (s, 3H, H-7), 1.80 (dd, 2H, H-11), 1.50 (m, 2H, H-10), 1.30 (d, 3H, H-9), 1.24 (s, 3H, H-14), 1.21 (s, 3H, H-15); ^{13}C NMR ($CDCl_3$/TMS) (δ/ppm): 187.30 (C-1), 184.20 (C-4), 151.20 (C-3), 140.80 (C-5), 135.80 (C-6), 122.10 (C-2), 61.50 (C-12), 49.00 (C-13), 31.20 (C-10), 29.50 (C-8), 26.40 (C-11), 23.20 (C-14), 18.40 (C-15), 18.00 (C-9), 14.70 (C-7); EIMS (70 eV) *m/z* (%): 264 (12) $M^{+\bullet}$, 206 (80) $[M-58]^{+\bullet}$, 191 (57) $[M-19]^{+}$, 177 (58) $[M-87]^{+}$, 167 (100) $[M-97]^{+}$, 166 (35) $[M-98]^{+\bullet}$; HRMS-EI (70 eV): elemental composition of $C_{15}H_{20}O_4$ for molecular ion, exact value 264.1364 Da, and precise value 264.1355 Da, with error of -2.9 ppm, and complementarily, the provided unsaturations data of 6.0.

2-[4-(3,3-dimethyloxiran-2-yl)butan-2-yl]-6-hydroxy-5-methylcyclohexa-2,5-diene-1,4-dione (**4**): yellow solid was obtained with 65.4% isolated yield; mp 84-86 °C; ^{1}H NMR ($CDCl_3$/TMS) (δ/ppm): 7.18 (bs,1H, OH), 6.47 (s, 1H, H-3), 2.96 (m, 1H, H-8), 2.70 (dd, 1H, H-12), 1.94 (s, 3H, H-7), 1.75-1.50 (m, 4H, H-10 and H-11), 1.16 (d, 3H, H-9), 1.24 (s, 3H, H-14), 1.30 (s, 3H, H-15); ^{13}C NMR ($CDCl_3$/TMS) (δ/ppm): 188.40 (C-1), 183.10 (C-4), 133.10 (C-3), 116.90 (C-5), 151.60 (C-6), 149.50 (C-2), 56.40 (C-12), 49.00 (C-13), 31.70 (C-10), 31.20 (C-8), 23.10 (C-11), 26.40 (C-14), 19.60 (C-15), 18.00 (C-9), 7.80 (C-7); EI-MS (70 eV) *m/z* (%): 264 (20) $M^{+\bullet}$, 247 (19) $[M-17]^{+}$, 193(17) $[M-71]^{+}$, 167 (100) $[M-97]^{+}$, 138 (8) $[M-126]^{+\bullet}$; HRMS-EI (70 eV): elemental composition of $C_{15}H_{20}O_4$ for molecular ion, exact value 264.1364 Da, and precise value 264.1362 Da for molecular ion, and precise value 264.1371 Da, with error of +3.7 ppm, and complementarily, the provided unsaturations data of 6.0.

3-hydroxy-5-methyl-2-[4-(2,2,5,5-tetramethyl-1,3-dioxolan-4-yl)butan-2-yl]cyclohexa-2,5-diene-1,4-dione (**5**): yellow solid was obtained with 50.2% isolated yield; mp 74-75 °C; ^{1}H NMR ($CDCl_3$/TMS) (δ/ppm): 6.47 (s, 1H, H-6), 3.64 (t, 1H, H-12), 3.17(bs, 1H, OH), 3.09 (m, 1H, H-8), 2.1 (s, 3H, H-7), 1.49-1.32 (m, 4H, H-10 and H-11), 1.27-1.26 (s, 6H, H-17 and H-18), 1.22 (s, 3H, H-14), 1.21 (s, 3H, H-15), 0.96 (d, 3H, H-9). ^{13}C NMR ($CDCl_3$/TMS) (δ/ppm): 188.10 (C-1), 185.20 (C-4), 153.80 (C-3), 141.90 (C-5), 124.10 (C-2), 135.80 (C-6), 106.70 (C-16), 84.10 (C-12), 80.50 (C-13), 31.90 (C-10), 30.50 (C-8), 28.80 (C-17 and C-18), 28.50 (C-11), 27.20 (C-14), 26.50 (C-15), 23.20 (C-9), 14.70 (C-7); EI-MS (70 eV) *m/z* (%): 322 (not observed) $M^{+\bullet}$, 307 (100) $[M-15]^{+}$, 265 (80) $[M-57]^{+}$, 247 (58) $[M-75]^{+}$, 206 (75) $[M-116]^{+\bullet}$, 177 (66) $[M-145]^{+}$, 167 (58) $[M-155]^{+}$; HRMS-EI (70 eV): elemental composition of $C_{17}H_{23}O_5$ for the fragment *m/z* 307, exact value 307.1545 Da, and precise value 307.1539 Da, with error of -2.0 ppm, and complementarily, the provided unsaturations data of 6.5.

6-hydroxy-5-methyl-2-[4-(2,2,5,5-tetramethyl-1,3-dioxolan-4-yl)butan-2-yl]cyclohexa-2,5-diene-1,4-dione (**6**): yellow solid was obtained with 77.4% isolated yield; mp 102-103 °C: ^{1}H NMR ($CDCl_3$/TMS) (δ/ppm): 6.46 (s, 1H, H-3), 3.66 (t, 1H, H-12), 2.94 (m, 1H, H-8), 1.84 (s, 3H, H-7), 2.10 (b, 1H, OH), 1.70 (m, 4H, H10 and

H-11), 1.30 (s, 3H, H-17) 1.24 (s, 3H, H-18), 1.17 (s, 3H, H-14), 1.00 (s, 3H, H-15), 1.16 (d, 3H, H-9); ^{13}C NMR ($CDCl_3$/TMS) (*δ*/ppm): 188.40 (C-1), 184.10 (C-4), 154.10 (C-6), 150.80 (C-2), 133.30 (C-3), 116.80 (C-5), 106.80 (C-16), 83.90 (C-12), 80.50 (C-13), 33.60 (C-10), 32.50 (C-8), 28.80 (C-17), 27.40 (C-11), 27.10 (C-18), 26.40 (C-14), 23.20 (C-9), 19.70 (C-15), 7.80 (C-7); EI-MS (70 eV) *m/z* (%): 322 (not observed) $M^{+\bullet}$, 307 (100) $[M-15]^{+}$, 265 (41) $[M-57]^{+}$, 247 (6) $[M-75]^{+}$, 206 (32) $[M-116]^{+\bullet}$, 194 (66) $[M-131]^{+}$, 167 (27) $[M-155]^{+}$; HRMS-EI (70 eV): elemental composition of $C_{17}H_{23}O_5$ for the fragment *m/z* 307, exact value 307.1545 Da, and precise value 307.1545 Da, with error of -2.0 ppm, and complementarily, the provided unsaturations data of 6.5.

3-hydroxy-6-(2-hydroxy-4-methyl-3,6-dioxocyclohexa-1,4-dien-1-yl)-2-methylheptan-2-yl acetate (**7**): red oil was obtained with 34.7% isolated yield; ^{1}H NMR ($CDCl_3$/TMS) (*δ*/ppm): 7.20 (b, 2H, OH-3, OH-12), 6.48 (s, 1H, H-6), 4.8 (dd, 1H, H-12), 3.05 (m, 1H, H-8), 2.10 (s, 3H, H-17), 2.06 (s, 3H, H-7), 1.98-1.34 (m, 4H, H-10 and H-11), 1.20 (d, 3H, H-9), 1.15 (s, 6H, H-14 and H-15); ^{13}C NMR ($CDCl_3$/TMS) (*δ*/ppm): 187.30 (C-1), 184.20 (C-4), 171.20 (C-16), 151.20 (C-3), 140.70 (C-5), 135.70 (C-6), 123.90 (C-2), 79.70 (C-12), 72.30 (C-13), 30.40 (C-10), 29.10 (C-8), 27.90 (C-11), 26.50 (C-14), 24.90 (C-15), 21.00 (C-17), 14.70 (C-7), 18.20 (C-9). EI-MS (70 eV) *m/z* (%): 324 (1) $M^{+\bullet}$, 224 (100) $[M-100]^{+\bullet}$, 309 (2) $[M-15]^{+}$, 296 (4) $[M-28]^{+\bullet}$, 278 (8) $[M-46]^{+\bullet}$, 264 (7) $[M-60]^{+\bullet}$; HRMS-EI (70 eV): elemental composition of $C_{17}H_{24}O_6$ for molecular ion, exact value 324.1573 Da, and precise value 324.1601 Da, with error of +8.8 ppm, and complementarily, the provided unsaturations of 6.0.

3-hydroxy-6-(5-hydroxy-4-methyl-3,6-dioxocyclohexa-1,4-dien-1-yl)-2-methylheptan-2-yl acetate (**8**): yellow solid was obtained with 28% isolated yield; mp 110-112 °C; ^{1}H NMR ($CDCl_3$/TMS) (*δ*/ppm): 7.02 (b, 2H, OH-6 and OH-12), 6.43 (s, 1H, H-3), 4.80 (dd,1H, H-12), 2.89 (m, 1H, H-8), 2.12 (s, 3H, H-17), 1.94 (s, 3H, H-7), 1.70-1.40 (m, 4H, H-10 and H-11), 1.13 (d, 3H, H-9), 1.17 (s, 6H, H-14 and H-15); ^{13}C NMR ($CDCl_3$/TMS) (*δ*/ppm): 188.00 (C-1), 183.10 (C-4), 171.10 (C-16), 151.40 (C-6), 149.00 (C-2), 133.20 (C-3), 116.90 (C-5), 79.20 (C-12), 72.30 (C-13), 32.00 (C-10), 31.60 (C-8), 27.10 (C-11), 26.50 (C-14), 25.20 (C-15), 21.00 (C-17), 18.70 (C-9), 7.80 (C-7); EI-MS (70 eV) *m/z* (%): 324 (1) $M^{+\bullet}$, 224 (100) $[M-100]^{+\bullet}$, 306 (26) $[M-18]^{+\bullet}$, 264 (30) $[M-60]^{+\bullet}$, 236 (5) $[M-28]^{+\bullet}$; HRMS-EI (70 eV): elemental composition of $C_{17}H_{24}O_6$ for molecular ion, exact value 324.1573 Da, and precise value 324.1601 Da, with error of +6.3 ppm, and complementarily, the provided unsaturations data of 6.0.

2-(5,6-Dihydroxy-6-methylheptan-2-yl)-3-hydroxy-5-methylcyclohexa-2,5-diene-1,4-dione (**9**): purple oil was obtained with 50% isolated yield; ^{1}H NMR ($CDCl_3$/TMS) (*δ*/ppm): 7.25 (b, 2H, OH-12 and OH-13), 6.75 (b, 1H, OH-3), 6.49 (s, 1H, H-6), 3.40 (dd, 1H, H-12), 3.07 (m, 1H, H-8), 2.10 (s, 3H, H-7), 1.5-2.0, (m, 4H, H-10 and H-11), 1.22 (s, 3H, H-15), 1.16 (s, 3H, H-14), 1.13 (s, 3H, H-9); ^{13}C NMR ($CDCl_3$/TMS) (*δ*/ppm): 187.77 (C-4), 184.24 (C-1), 151.09 (C-3), 140.90 (C-5), 135.59 (C-6), 124.14 (C-2), 78.66 (C-12), 77.34 (C-13), 30.99 (C-10), 29.69 (C-8), 28.70 (C-11), 26.33 (C-14), 22.97 (C-9), 18.23 (C-15), 14.03 (C-7); EIMS (70 eV)

m/z (%): 282 (3) $M^{+\bullet}$, 167 (100) $[M-115]^+$, 264 (7) $[M-18]^{+\bullet}$, 224 (12) $[M-58]^{+\bullet}$, 206 (10) $[M-76]^{+\bullet}$, 185 (28) $[M-97]^+$, 166 (15) $[M-116]^{+\bullet}$; HRMS-EI (70 eV): elemental composition of $C_{15}H_{22}O_5$ for molecular ion, exact value 282.1467 Da, and precise value 282.1472 Da, with error of +1.6 ppm, and complementarily, the provided unsaturations data of 5.0.

2-(5,6-Dihydroxy-6-methylheptan-2-yl)-6-hydroxy-5-methylcyclohexa-2,5-diene-1,4-dione (**10**): yellow solid was obtained with 50% isolated yield; mp 112-114 °C; ^{1}H NMR ($CDCl_3$/TMS) (δ/ppm): 7.5 (b, 2H, OH-12 and OH-13), 7.2 (b, 1H, OH-6), 6.48 (s, 1H, H-3), 3.40 (dd, 1H, H-12), 2.92 (m, 1H, H-8), 1.9 (s, 3H, H-7), 1.7-1.40 (m, 4H, H-10 and H-11), 1.23 (s, 3H, H-15), 1.21 (s, 3H, H-14), 1.17 (d, 3H, H-9); ^{13}C NMR ($CDCl_3$/TMS) (δ/ppm): 188.30 (C-1), 183.40 (C-4), 151.70 (C-6), 149.35 (C-5), 133.10 (C-3), 117.10 (C-2), 77.50 (C-12), 72.50 (C-13), 31.23 (C-10), 28.90 (C-8), 26.46 (C-11), 23.29 (C-14), 19.62 (C-9), 18.80 (C-15), 7.87 (C-7); EIMS (70 eV) *m/z* (%): 282 (5) $M^{+\bullet}$, 264 (10) $[M-18]^{+\bullet}$, 224 (22) $[M-58]^{+\bullet}$, 206 (13) $[M-76]^{+\bullet}$, 193 (17) $[M-89]^+$, 167 (100) $[M-115]^+$, 166 (20) $[M-116]^{+\bullet}$; HRMS-EI (70 eV): elemental composition of $C_{15}H_{22}O_5$ for molecular ion, exact value 282.1467 Da, and precise value 282.1444 Da, with error of -8.2 ppm, and complementarily, the provided unsaturations data of 5.0.

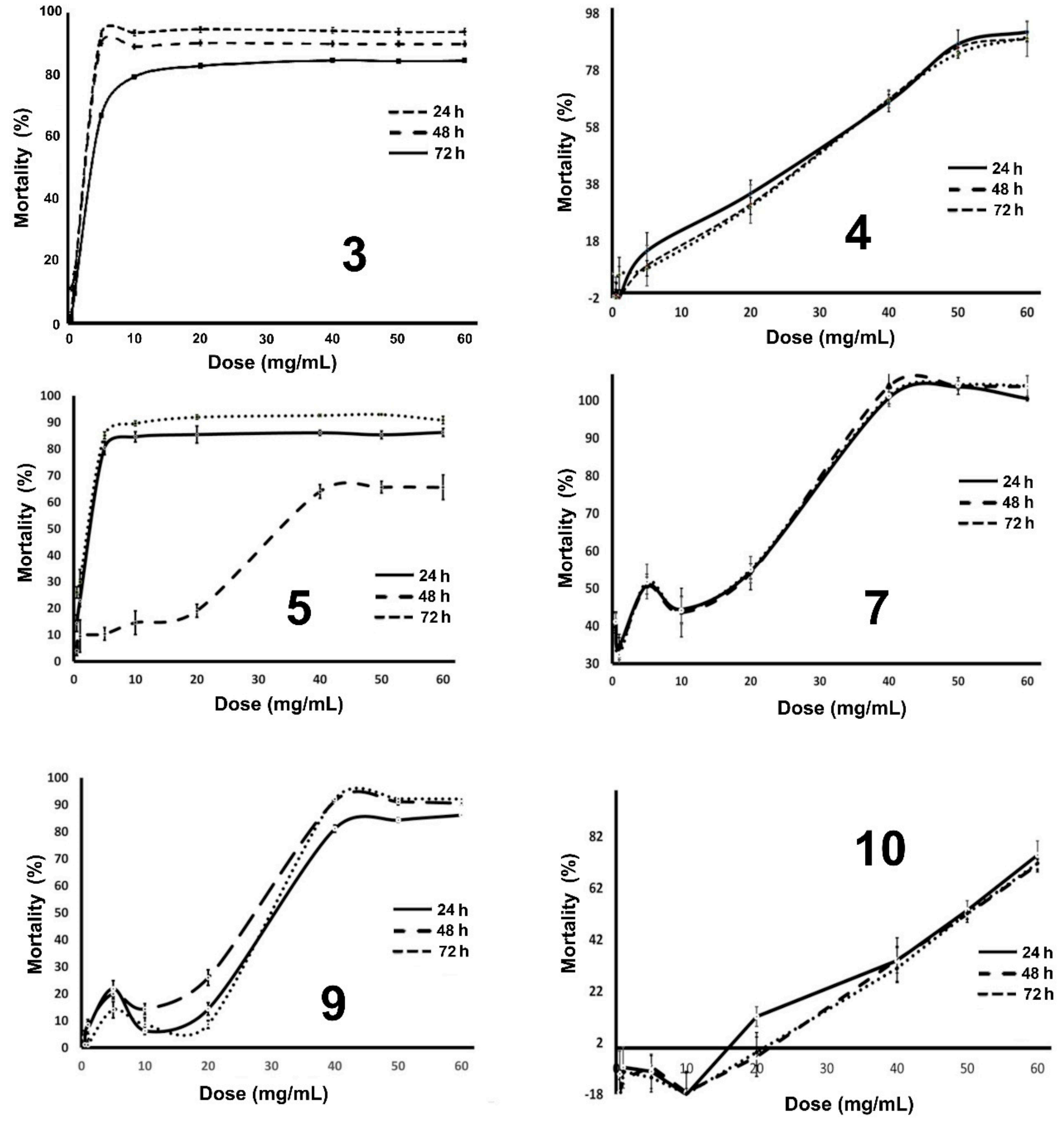

Figure S1. Dose-response curves for cytotoxicity on breast cancer cells of derivatives **3-5**, **7**, **9-10**.

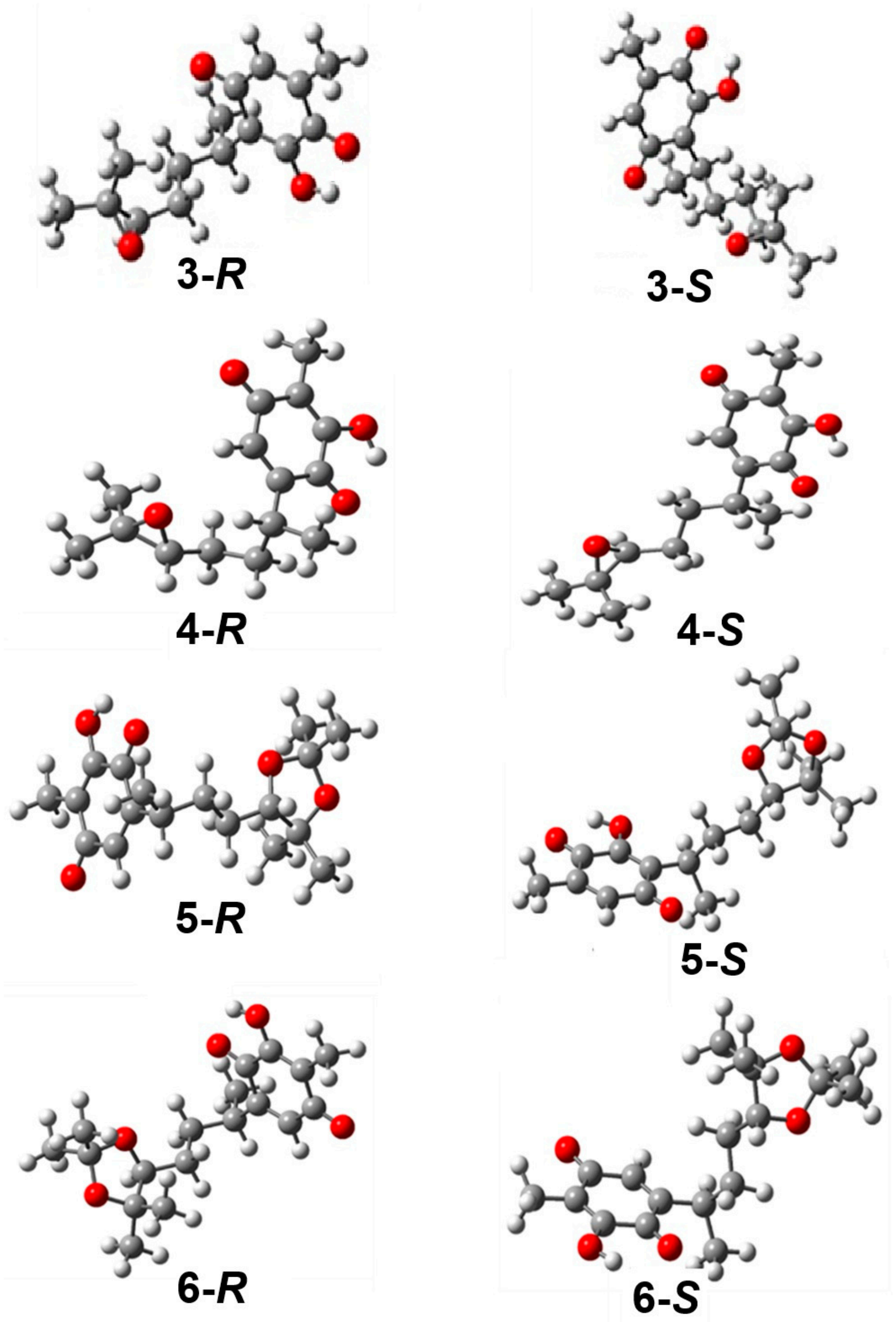

Figure S2. Optimized geometries for **3-6** with their respective stereocenter at C-12.

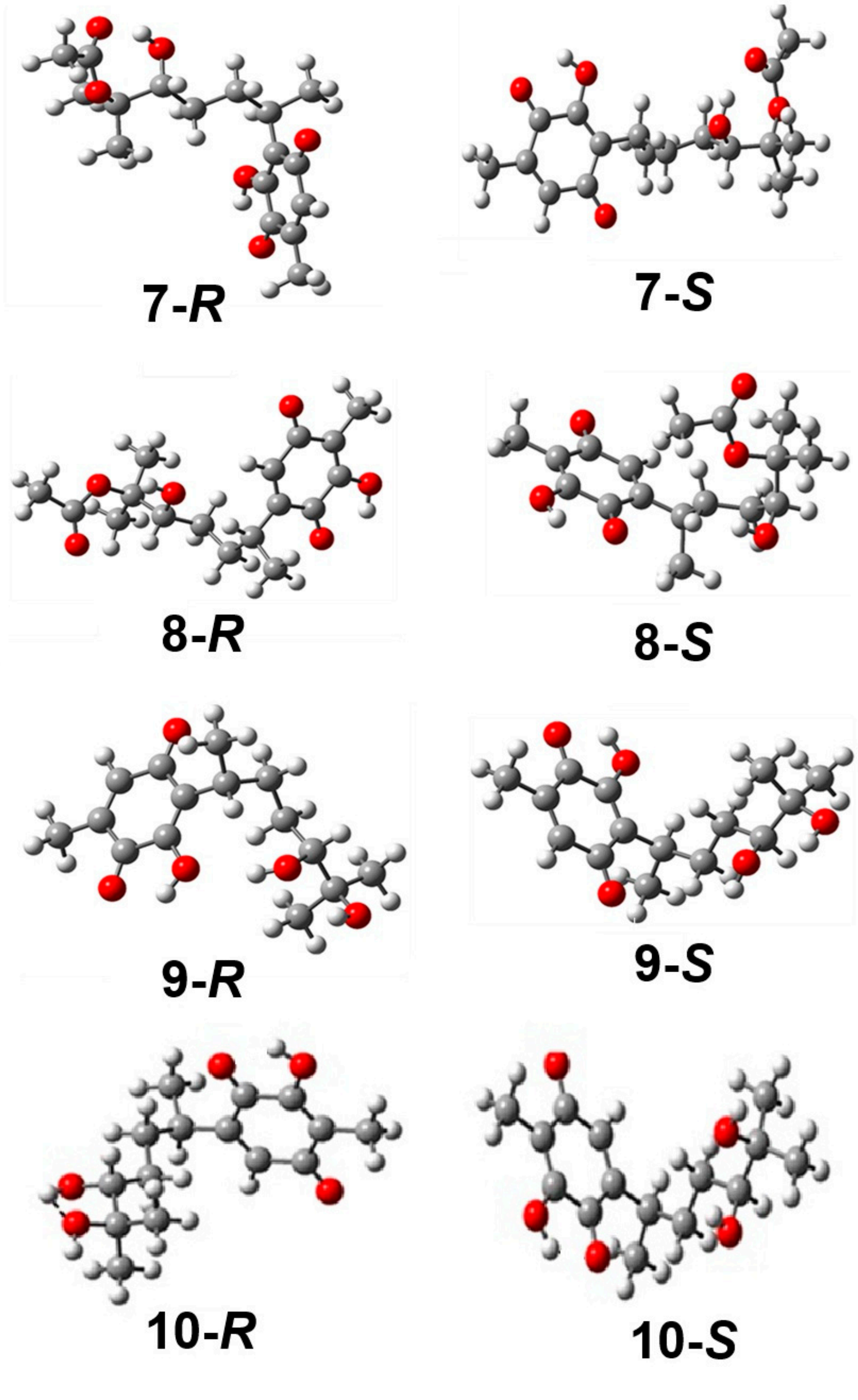

Figure S3. Optimized geometries for **7-10** with their respective stereocenter at C-12.

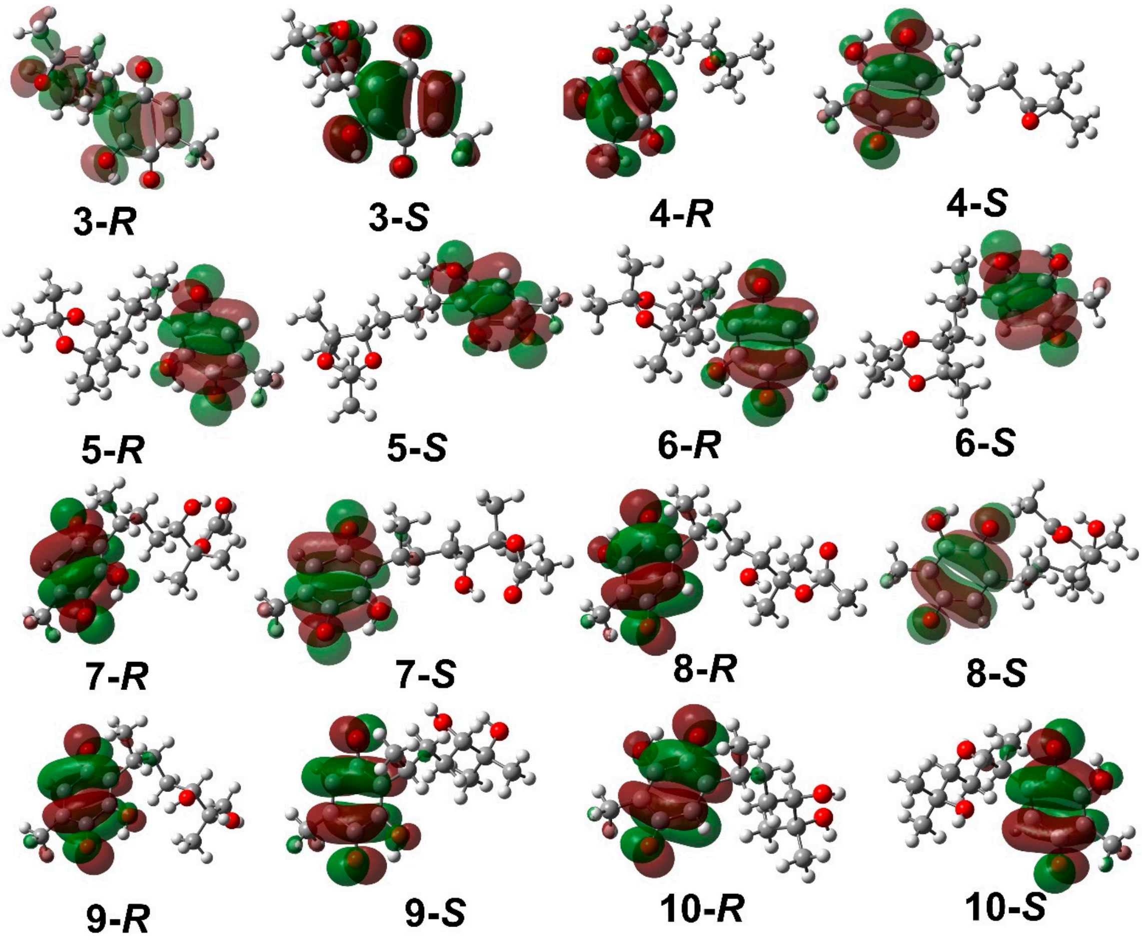

Figure S4. LUMO molecular orbital of the studied molecules.

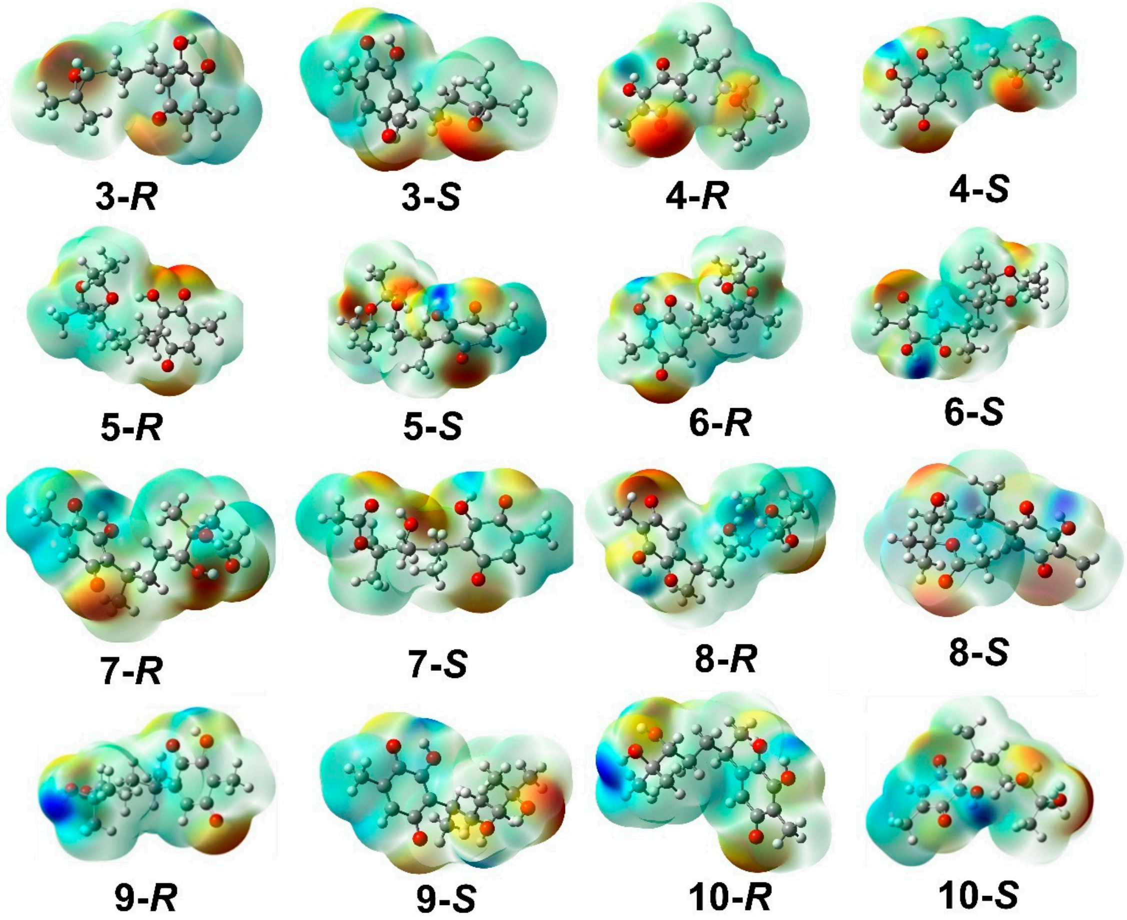

Figure S5. Molecular electrostatic potential maps for stereoisomers with lower E_{LUMO} of compounds **3-10**.

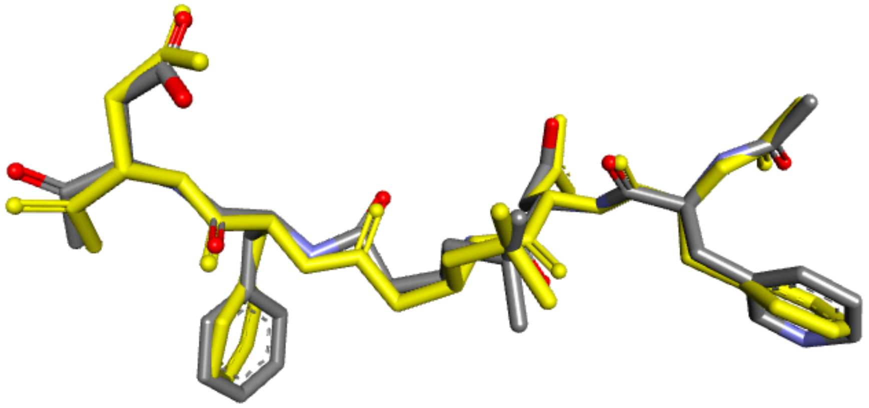

Figure S6. RMSD for co-crystallized ligand Ac-DW3-KE (yellow) with respect to reference ligand at the crystal structures (gray) for illustrating good docking solution (RMSD ≤ 2.0 Å).
